# A Preliminary Qualitative Analysis of women's Experiences With Vaginal Fractional CO_2_ Laser Treatments

**DOI:** 10.1093/asjof/ojae074

**Published:** 2024-09-03

**Authors:** Anne Nileshni Fernando, Christine Hamori, Jayson Oates, Gemma Sharp

## Abstract

**Background:**

Menopause involves a range of bodily changes, with impacts on physical and psychological well-being. Around half of the postmenopausal women experience genitourinary syndrome of menopause (GSM). Fractional CO_2_ laser treatment can promote tissue regeneration in the vaginal wall to potentially assist with managing GSM. However, the results from clinical trials of this treatment have been mixed, and the personal perceptions and experiences of women receiving this treatment have been largely unexplored.

**Objectives:**

To qualitatively explore the motivations and outcomes of women who have undergone vaginal fractional CO_2_ laser treatment.

**Methods:**

Fourteen postmenopausal women were involved in the study. These women had undergone vaginal fractional CO_2_ laser treatment between 2 and 48 months earlier (*M* = 32.1, standard deviation = 14.9 months). Telephone interviews were conducted to explore women's motivations and their experiences after treatment. Interviews were recorded and transcribed verbatim. Deductive and inductive thematic analysis was conducted to analyze interviews.

**Results:**

Analyses produced 4 major themes. First, motivations mostly revolved around participants seeking relief from menopausal symptoms. Second, some participants noted positive sexual outcomes, including improved sexual pleasure after treatment. Third, participants noted positive physical and psychological effects, including improvements in incontinence and overall confidence. Lastly, a subset of participants reported no discernible changes.

**Conclusions:**

This novel qualitative exploration of women's motivations and outcomes of fractional CO_2_ laser therapy demonstrates the multifaceted impact of treatment. These findings highlight the importance of considering the holistic effects of fractional CO_2_ laser therapy on women's health during midlife, particularly amid menopausal changes.

**Level of Evidence: 4:**

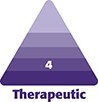

Menopause can be a time of profound change to the mind and body. Genitourinary syndrome of menopause (GSM) encompasses a collection of symptoms resulting from hormonal changes accompanying menopause. These symptoms include dyspareunia, itching, burning pain, recurrent vaginal infections, urinary incontinence, and urinary tract infecitons.^1,2^ These symptoms affect approximately half of all postmenopausal women and have been reported to negatively impact physical comfort, sexual function, social relationships, and quality of life.^3^

At present, symptoms are typically managed using hormonal therapies such as topical estrogen or nonhormonal vaginal lubricants and moisturizers aimed at providing symptomatic relief.^3^ Although research supports the use of topical vaginal estrogen for symptoms such as vaginal dryness, burning, or dyspareunia,^4^ women frequently express concerns about using hormonal products of any kind on or in their bodies fearing they might interfere with the body's natural processes.^5^ Fractional CO_2_ laser therapy has emerged as a nonhormonal intervention for postmenopausal genitourinary symptoms, although evidence supporting its effectiveness is mixed according to systematic reviews.^3,6^ Currently, in the United States and Australia, laser therapies have not been approved for treatment of menopausal symptoms because of limited high-quality research, supporting long-term benefits and safety.^7,8^

For instance, a randomized controlled trial (RCT) showed no significant differences in improvements to GSM symptoms or quality of life between those who underwent fractional CO_2_ therapy treatment (*n* = 43) and those undergoing a sham laser procedure (*n* = 42) at 12 months postprocedure.^9^ Although a number of commentaries (eg, Ngenda^10^) have queried that the treatment protocol employed in this RCT may have been atypical. Nevertheless, another RCT demonstrated no significant differences in symptoms of GSM as well as sexual and urinary function after 6 months for women who underwent fractional CO_2_ vaginal laser treatment (*n* = 30) and those who received vaginal estrogen (*n* = 32).^11^ Interestingly, within this study, 79% of participants who received fractional CO_2_ laser therapy reported being “satisfied or very satisfied,” compared with 73% who received vaginal estrogen, a difference that was not statistically significant.^11^ In contrast, a systematic review and meta-analysis of RCTs found greater improvements in vaginal elasticity, epithelium, pH, moisture, as well as secretion fluid volume and consistency in women who underwent fractional CO_2_ laser therapy compared with those who received a sham laser therapy.^1^ This review, however, only included 3 studies (with 233 participants in total) and highlighted the need for further high-quality, large scale, and long-term studies to determine the extent of long-term effects of the procedure.^1^ Additionally, another prospective study observing postmenopausal women (*n=* 77*)* reported improvements to GSM symptoms in women who had undergone fractional CO_2_ laser treatments with significant improvements to sexual function and quality of life.^12^ Importantly, improvements in vaginal and urinary health as well as sexual function have been found to be sustained at 12 months.^13^

Although RCTs and observational studies appear to present some conflicting data on the outcomes of fractional CO_2_ laser therapy, there is a notable absence of in-depth qualitative explorations of the individual experiences of fractional CO_2_ laser therapy. Qualitative explorations in research can offer valuable, nuanced insights into experiences, emotions, and perceptions surrounding the procedure which cannot be captured in quantitative questionnaires. For example, novel findings have previously emerged from qualitative studies exploring experiences of women who had undergone labiaplasty, particularly that many women are concerned about sexual partners noticing scarring on their labia minora and potentially mistaking it for genital gender affirming surgery.^14,15^ Although research has shown that this concern is generally not found in reality with both people in the community as well as aesthetic surgeons not being able to decipher whether a female has or has not undergone labiaplasty from images of their vulva,^16^ such a clinically relevant finding would not have been possible using standardized quantitative measures. As a result of these qualitative findings, aesthetic surgeons can allay women's concerns about scarring before labiaplasty. Thus, the aim of the current study was to qualitatively explore, for the first time, the motivations and outcomes of women undergoing vaginal CO_2_ fractional laser therapy and provide new in-depth insights which could have implications for clinical practice.

## METHODS

### Participants

Participants were 14 postmenopausal women aged 36 to 68 years (*M* = 52.2, standard deviation [SD] = 9.7 years). Menopause was reached either through the natural aging process or was medically induced earlier in life through treatment for cancer. Participants were eligible to participate in the study if they were a cisgender postmenopausal female who had undergone at least 1 fractional CO_2_ laser treatment session in the last 4 years and were proficient in the English language. Four years was chosen to increase the diversity in potential outcomes and add to the richness of the qualitative data collected. The study was approved by the Monash University Human Research Ethics Committee and was part of a wider study described elsewhere.^14,15^

### Procedure

Participants were recruited from 2 private plastic surgery clinics employing the same treatment protocol (see Samuels et al^17^ and Athanasiou et al^18^ for specific details). Recruitment of this sample has been reported previously.^14,15^ Briefly, clinic reception staff contacted and provided study information (through email, mail, and phone) to potential participants who had undergone CO_2_ laser therapy in the last 4 years. The treating clinicians, authors C.H. and J.O., were not involved in the contact of any participants. Interested participants were directed to contact the study lead G.S. to organize a suitable time to conduct a telephone interview. Informed consent was obtained in written form before the interview and verbally at the start of the interview. Interviews were conducted between January and April 2019. All interviews were conducted by author G.S. The interviewing author was not involved in any aspect of the treatment of the participant.

### Interviews

Semistructured qualitative interviews were conducted through telephone and lasted between 12 and 55 min (*M* = 28.1; SD = 12.5). Interview questions were developed by the research team which included researchers, psychologists, and aesthetic surgeons and are provided as an Appendix. The questions were designed to conduct an in-depth exploration of the participants’ motivations to undergo fractional CO_2_ laser therapy as well as experiences, emotions, and perspectives of the treatment outcomes. All interviews were audio recorded and transcribed verbatim. Interviews were conducted until author A.N.F., in discussion with co-authors, detected saturation. This point was identified when no new themes, codes, or insights were identified in the data indicating that additional interviews would not yield further themes.^19,20^ This study provided a moderate-to-large sample size for qualitative research.^19,20^ Our analysis approach involved a hybrid deductive and inductive thematic analysis methodology, as described by Braun and Clarke.^21^ In concordance with this approach, the primary analyst A.N.F. initially familiarized herself with the transcripts, and deductively coded the transcripts according to the semistructured interview questions. This was followed by an inductive approach, developing codes as they emerged through the data. The primary analyst A.N.F. examined all transcripts and discussed initial coding with a secondary analyst G.S. Together the primary and secondary analysts generated themes and subthemes that were relevant to the aims of this project.

## RESULTS

Fourteen postmenopausal women aged 36 to 68 years (*M* = 52.2, SD = 9.7 years) were interviewed to explore motivations and experiences after undergoing fractional CO_2_ laser therapy. The women were between 2 and 48 months postfractional CO_2_ laser therapy (*M* = 32.1, SD = 14.9 months). The number of fractional CO_2_ laser treatments varied with participants receiving 1 treatment (*n* = 1, 7.1%), 2 treatments (*n* = 1, 7.1%), 3 treatments (*n* = 7, 50.0%), 4 treatments (*n* = 4, 28.6%), or 6 treatments (*n* = 1. 7.1%). None of the participants explicitly reported any complications. The major themes and subthemes from the interviews are described below. Participant demographic characteristics are outlined in Table 1.

**Table 1.** Participant Demographics Characteristics (*n* = 14)

Characteristic	*n* (%)
Age (years), mean (SD)	52.2 (9.7)
Range	36-68
Ethnicity	
White	13 (92.9)
Hispanic	1 (7.1)
Sexual orientation	
Heterosexual	14 (100.0)
Current romantic relationship	
Yes	8 (57.1)
No	6 (42.9)
Children	
Yes	13 (92.9)
No	1 (7.1)
Highest education achieved	
High school	2 (14.3)
TAFE/diploma	2 (14.3)
Undergraduate degree	7 (50.0)
Postgraduate degree	3 (21.4)

SD, standard deviation.

### Motivations and Perceptions of Procedure

#### Onset of Menopausal Symptoms

There were a variety of reasons reported by participants for pursuing fractional CO_2_ laser therapy. Most of the reasons were related to addressing symptoms of GSM (*n* = 12, 85.7%), such as physical discomfort during sexual intercourse (*n* = 6, 42.9%), incontinence (*n* = 5, 35.7%), dryness (*n* = 4, 28.6%), and frequent urinary tract infections (*n* = 1, 7.1%).

“Having gone through menopause, you’re aware that you get drier and I was worried about the stress incontinence that was getting worse so I was looking for options to address these symptoms.” (Participant 4, aged 65 years, 4 treatments, 48 months post final treatment)

Participants also expressed frustration at the lack of treatment options to address symptoms of GSM.

“It's just more frustrating, ‘why isn’t there more things out there for women?’… because we all go through it [menopause]! And there are a lot of women who are just embarrassed, won’t talk about it and they just don’t have sex anymore and I think it's just so wrong! It's just not right!” (Participant 9, aged 57 years, 6 treatments, 40 months postfinal treatment)

#### Alternate Treatments to Hormones

In navigating menopausal symptoms, some participants (*n* = 4, 28.6%) discussed pursuing alternative treatments to hormonal therapies expressing reluctance to use “unnatural” hormonal products.

“I tried a non-hormonal, medicated vaginal cream, then they gave me this hormone cream and it's not really helping much and then I was worried about the hormones and I thought well why don’t I try to fix it rather than put a band-aid on it.” (Participant 5, aged 62 years, 3 treatments, 12 months postfinal treatment)

#### Perceptions of Procedure

The minimally invasive nature of the procedure as well as the absence of recovery time seemed to resonate with the participants (*n* = 3, 21.4%), emerging as a viable and convenient option to pursue (*n* = 3, 21.4%).

“It's non-invasive, minimal down-time, that's a selling point—hugely.” (Participant 1, aged 41 years, 3 treatments, 2 months postfinal treatment)

However, participants (*n* = 3, 21.4%) encountered varying reactions from health professionals (mostly gynecologists). Some were unaware of the procedure, whereas some expressed varying opinions about the procedure.

“I said to the gynecologist, ‘…I’ve had the [fractional CO_2_ laser therapy] and I thought he did a bit of an eye-roll…’ some doctors you can go and say, ‘…I’m having acupuncture’ and they’ll go, ‘…that's really good’ or they’ll go, ‘…you’re wasting your money.’” (Participant 8, aged 51 years, 3 treatments, 48 months postfinal treatment)

Despite these encounters, a proportion of participants (*n* = 2, 14.3%) believed that more health professionals should be aware of this procedure and its potential impacts on women navigating menopausal symptoms.

“He [primary care professional] may be able to suggest this when there aren’t any other avenues for women.” (Participant 9, 57 years of age, 6 treatments, 40 months postfinal treatment)

### Positive Sexual Outcomes

Almost half of participants (*n* = 6, 42.9%) reported improvements in their sexual experiences post treatment. They specifically noted the absence of pain and an increase in pleasurable sensations. Additionally, some participants (*n* = 3, 21.4%) attributed these enhanced sexual experiences to improvements in confidence during sexual encounters. Improved lubrication during intercourse because of being able to enjoy the experience posttreatment was also reported by a proportion of these participants (*n* = 2, 14.3%). One participant highlighted the change in sexual activity with her partner and the effect on their relationship.

“We were able to have longer sex. My husband didn’t want to have sex because he was worried it was hurting me. I said, ‘I’m okay, I’m okay.’ It helped the relationship that way as well. As I say, it's just feeling better within myself that I was able to have sex again and put his mind at ease that it didn’t hurt anymore.” (Participant 9, aged 57 years, 6 treatments, 40 months postfinal treatment)

Moreover, a few participants (*n* = 2, 14.3%) indicated interest in pursuing additional treatments because of perceived short-term results in sexual function.

“Yes, you feel a little bit better for a while, but I will say that after repetitive…sexual intercourse and months going by, you go back to where you were.” (Participant 6, aged 68, 3 treatments, 36 months postfinal treatment)

### Positive Physical and Psychological Well-being Outcomes

Participants noted improvements to incontinence concerns (*n* = 3, 21.4%). Two participants reported that by addressing incontinence concerns, the procedure had facilitated continued engagement in physical activity (eg, running) which was reported to improve their quality of life.

“I didn’t originally go in there from a cosmetic look or for a sexual interest issue. It was more like I said, to take care- increase my quality of my running and try to take care of that issue. And it has helped.” (Participant 3, aged 55 years, 3 treatments, 21 months postfinal treatment)

Additionally, participants (*n* = 6, 42.9%) reported improvements to psychological well-being following the procedure, specifically improving confidence and self-esteem.

“You do definitely have a difference in your psychological wellbeing. I think you do feel a lot more confident and comfortable… you feel a lot better about yourself.” (Participant 11, aged 60 years, 4 treatments, 42 months postfinal treatment)

### Limited or Lack of Outcomes

Some participants reported that the procedure did not meet specific expectations with a proportion of participants (*n* = 3, 21.4%), reporting no major changes after treatment.

“I had three [treatments] I think. Because I remember after the second [treatment] I still wasn’t happy and it was like nothing! I think they gave me three and there was nothing.” (Participant 7, aged 40, 3 treatments, 36 months postfinal treatment)

There were some reports however (*n* = 2, 14.3%) of observing changes only after multiple sessions suggesting that 1 treatment may not suffice.

“After the procedure I was hoping that it would be more of a better—I guess a better experience sexually after the first treatment, I guess. I really didn’t notice a whole difference. It was really the second and third, I really started to feel different.” (Participant 2, aged 36 years, 3 treatments, 24 months postfinal treatment)

Interestingly, a minority remained unaffected by changes in specifically sexual pleasure posttreatment.

“But as far as the tightening and that kind of stuff or the other thing is I noticed that one of the things women talk about is it's easier for them to climax after they get this done. I didn’t notice the difference.” (Participant 2, aged 36 years, 3 treatments, 24 months postfinal treatment)

## DISCUSSION

To the authors’ knowledge, this study represents the first preliminary qualitative exploration of motivations and outcomes associated with vaginal fractional CO_2_ laser therapy. Although a range of quantitative studies have suggested moderate–to-high patient satisfaction rates,^6,11^ our qualitative approach allowed for an in-depth analysis of women's experiences, including reasons underlying these simpler quantitative results. Our study highlighted that most women pursued this treatment to address the onset of symptoms linked to GSM, but also as a nonhormonal alternative. Additionally, our findings demonstrated the diverse range of experiences among women who underwent the procedure, with some reporting positive impacts across multiple facets of their life (ie, physical, psychological sexual), whereas others noted no discernible effects.

Many participants sought fractional CO_2_ laser therapy to manage menopausal symptoms (spontaneous or as a result of cancer and its treatment) associated with GSM. There was a proportion of women who were very opposed to any form of hormonal treatment for GSM because of concerns about the use of what they perceived to be “unnatural” hormones.^22^ Despite the potential benefits, fractional CO_2_ laser therapy has not received official approval for addressing menopausal symptoms.^7,8^ Yet, despite this lack of endorsement and varying perceptions and skepticism among healthcare professionals, women are pursuing fractional CO_2_ laser therapy. This may reflect a sense of frustration towards the lack of available and effective options to manage GSM symptoms (expressed in our sample) as well as desperation and urgency to address such symptoms in a way that existing hormonal treatments have not been able to. The lack of consensus among health professionals about fractional CO_2_ laser therapy may also reflect their uncertainty in effectively managing menopausal symptoms, despite their knowledge of menopause.^23^ Some women noted the procedure's noninvasive nature and the minimal recovery time required and believed that the procedure should be offered by health professionals to women who have exhausted other treatments. These factors combined with the reduced treatment frequency demands of fractional CO_2_ laser therapy compared with the frequent dosing of estrogen therapies, potentially served as appealing factors when considering pursuing the procedure.

Close to half of the women reported marked improvements in their sexual experiences following fractional CO_2_ laser therapy reflecting the diverse findings reported in previous clinical trial research.^6^ The women in our sample noted the need for continued treatment, owing to the return of symptoms such as vaginal laxity, dryness, and discomfort during sexual experiences after treatment. Improvements in sexual function after undergoing fractional CO_2_ laser therapy as measured by validated, quantitative measures, have been reported to persist for up to 18 months.^24^ However, there is limited research on outcomes, including sexual function outcomes beyond this period. Within our sample, there were reports of limited long-term benefits to sexual function from participants at 12 and 36 months postprocedure. Although we did not determine when effects began to diminish, this finding along with the mixed reports from the literature highlight the potential variation in individual experiences and the importance of perception.^13^

When exploring improvements to sexual experiences, several women reported enhancements in confidence during intimate encounters. It is possible that this improvement in confidence relates to the reported enhancements in psychological well-being by nearly half of the women. By alleviating the physical discomfort that women faced during sexual intercourse and improving lubrication, it is likely that they were able to feel more comfortable and confident in sexual encounters, contributing to more fulfilling experiences. Furthermore, improvements may also be linked to improved lubrication or changes to vaginal laxity and reduction in pain as reported by the women in our study and in existing literature.^17,25^ Interestingly, our study found limited outcomes with just 1 fractional CO_2_ laser therapy session, whereas existing research has reported the efficacy of fractional CO_2_ laser therapy for the management of GSM symptoms up to 12 months after treatment, regardless of the number of laser therapy sessions.^26^ Although not a focus of our study, particularly owing to its qualitative design, there were no obvious trends in outcomes based on number of treatments received or time since treatment. However, further research is needed.

A proportion of women reported no changes (physical, psychological, or sexual) after the procedure, even with multiple treatment sessions. This again reflects the mixed findings on the effectiveness of the procedure as some studies did not find notable vaginal symptom, urinary symptom, sexual function, and quality of life outcomes when compared with a control group (ie, sham laser therapy) or topical hormonal treatments.^3,11^ It is possible that menopausal status plays a role in the effectiveness of laser therapy. All of the women involved in our study were postmenopausal, and the research has suggested that early intervention with fractional CO_2_ laser therapy may be more effective in addressing menopausal symptoms. For example, 1 study found that improvements in vaginal health (ie, fluid volume, epithelial integrity, and moisture) were better sustained over time in recently postmenopausal women compared with those who >3 years postmenopausal.^13^ This further highlights the individual variation and the possible impact of menopausal changes, warranting further research.

This study has some limitations which should be considered. Although our sample captured diverse experiences following fractional CO_2_ laser therapy, it is likely biased toward participants who are more comfortable discussing their experiences, potentially excluding the motivations and experiences of those who are less comfortable or who have had less favorable outcomes. Given the sensitive nature of the topic, it is possible that some participants withheld details of certain experiences. Additionally, the sample included women at varying time points posttreatment, which may have impacted the accuracy of retrospective recall of specific motivations and outcomes. Moreover, we did not specifically ask participants to compare their experiences of the procedure to that of vaginal estrogen (if they had experienced this form of treatment), preventing direct comparisons. Despite these limitations, this qualitative approach contributes to our understanding of the diverse priorities and experiences of women undergoing fractional CO_2_ laser therapy. It also suggests the need for continued exploration and development of nonhormonal options that will effectively address symptoms of GSM.

## CONCLUSIONS

This study is the first qualitative exploration of motivations and outcomes associated with fractional CO_2_ laser therapy to address symptoms associated with GSM. Despite warnings by international societies against the use of vaginal fractional CO_2_ laser therapy because of limited supporting evidence, women pursued this procedure to address symptoms associated with GSM. Although most women reported significant improvements to GSM symptoms, sexual experiences, and psychological well-being, some women reported no changes in symptoms. These findings highlight the need for further research and development of nonhormonal treatment options for GSM.

## Supplementary Material

ojae074_Supplementary_Data
