# Supplementary material for: A Preliminary Qualitative Analysis of women's Experiences With Vaginal Fractional CO2 Laser Treatments
Source: Aesthet Surg J Open Forum. 2024 Sep 3;6:ojae074. doi: 10.1093/asjof/ojae074 (PMC11458914; doi:10.1093/asjof/ojae074)
Supplement: ojae074_Supplementary_Data [file ojae074_supplementary_data.docx]

**SUPPLEMENTARY MATERIAL**

**A preliminary qualitative analysis of women’s experiences with**

**vaginal fractional CO_2_ laser treatments**

**Fernando et al. (2024)**

**Semi-Structured Interview Guide:**

**Demographic characteristics:**

1. When did you have the vaginal fractional CO_2_ laser treatment(s)?
2. How many treatments did you have?
3. What is your current age?
4. How would you describe your ethnicity?
5. How would you describe your sexual orientation?
6. What is your current relationship status?
7. Do you have any children?
8. What is the highest level of education you have achieved?

**Pre- and post-treatment experiences (note that follow up questions were asked to clarify meaning etc given that these were semi-structured interviews):**

1. How would you describe your motivations for having vaginal fractional CO_2_ laser treatment?
2. Can you describe the results/effects of vaginal fractional CO_2_ laser treatment on physical health (e.g., stress incontinence)?
3. Can you describe the results/effects of vaginal fractional CO_2_ laser treatment on sexual function [if participant indicated they were sexually active]?
4. Can you describe the effects of vaginal fractional CO_2_ laser treatment on psychological wellbeing?
5. Did these effects meet your expectations?
